# Intrinsic attention to pain is associated with a pronociceptive phenotype

**DOI:** 10.1097/PR9.0000000000000934

**Published:** 2021-06-03

**Authors:** Greig Adams, Richard Harrison, Wiebke Gandhi, Carien M. van Reekum, Tim V. Salomons

**Affiliations:** a Department of Psychology and Clinical Language Sciences, University of Reading, Reading, United Kingdom; b Department of Psychology, Queen's University, Kingston, ON, Canada

**Keywords:** Intrinsic attention to pain, Temporal summation, Conditioned pain modulation, Pronociceptive phenotype

## Abstract

**Introduction:**

Evidence suggests that attention to pain is a product of both incoming sensory signals and cognitive evaluation of a stimulus. Intrinsic attention to pain (IAP) is a measure that captures an individual's natural tendency to attend to a painful stimulus and may be important in understanding why pain disrupts cognitive functioning in some individuals more than others.

**Objective:**

In this study, we explored the extent to which IAP was associated with the modulation of incoming sensory signals characteristic of a pronociceptive phenotype: temporal summation (TS) and conditioned pain modulation (CPM).

**Method:**

44 healthy participants (23 female; M_age_=23.57, S.D.=5.50) were assessed on IAP, TS and CPM.

**Results:**

We found that IAP was positively correlated with TS and CPM. A regression model showed that TS and CPM explained 39% of the variance in IAP scores. Both mechanisms seem to contribute independently to the propensity to attend to pain.

**Conclusion:**

These findings highlight that modulatory mechanisms at the spinal/supraspinal level exert a strong influence on an individual's ability to disengage from pain.

## Abstract 2

Capacity for modulation of incoming nociceptive signals is a determinant of our tendency to attend to pain.

## 1. Introduction

Individuals differ in their capacity to endogenously modulate nociceptive input. Yarnitsky et al.^21^ proposed a “pronociceptive phenotype” associated with nociceptive facilitation, consisting of enhanced temporal summation (TS), and/or less efficient conditioned pain modulation (CPM). Temporal summation is a measure of “wind-up” or enhancement of pain with prolonged nociceptive exposure, with accumulating intensity drawing increased attention to the stimulus, facilitating adaptive response. CPM is based on the concept of “pain inhibits pain” derived from diffuse noxious inhibitory control (DNIC) in animal studies.^10^ Conditioned pain modulation is said to quantify the efficiency of endogenous inhibition of pain.^4,14^ In Yarnitsky's conceptualisation, the 2 modulatory mechanisms contribute independently towards a pronociceptive phenotype, likely because of the ascending facilitatory component of TS^15,16^ and the descending inhibitory component of CPM.^9^ This suggests there are measurable individual differences in how people modulate an incoming nociceptive stimulus at the spinal and supraspinal level.

Pain is an alarming signal and naturally captures attention to facilitate adaptive avoidance of harm.^6^ The degree to which this focus is captured by pain may be a function of the competing salience of current contextual factors other than the nociceptive stimulus.^6^ However, attention to pain is not a simple function of pain intensity and the contextual factors external to the individual. Individuals differ in their trait-like ability to mentally disengage from pain when it occurs. A previous study found “intrinsic attention to pain” (IAP—the likelihood that people focus on pain or “something else” during nociceptive stimulation) was stable within individuals across time, with a high intraclass correlation across sessions, suggesting IAP can be considered trait-like. Furthermore, IAP was associated with their performance on a cognitive task during the presence of pain,^8^ suggesting this measure also taps individual differences in the likelihood that pain will distract from cognitive functioning. Neurally, IAP is associated with functional and structural connectivity between the medial prefrontal cortex and periaqueductal gray, suggesting an individual's propensity to attend to pain might be a function of the interplay between cortical evaluative processes and endogenous modulation of incoming sensory signals.

To date, however, little research has explored the role of the endogenous modulatory processes in how likely an individual is to be distracted by pain. This study, therefore, investigated whether (and how) a pronociceptive phenotype is associated with trait-like attention to pain.

## 2. Method

### 2.1. Participants

Forty-four healthy participants (23 female; M_age_ = 23.57, SD = 5.50) were recruited and received payment for their participation. The study was approved by the University of Reading Ethics Committee. Informed consent was provided by each participant. This study was part of an ongoing study involving 13 experimental sessions (1 sensory/cognitive assessment, 1 imaging session, and 11 examining prolonged pain exposure) per participant. All data for this study were collected during the initial sensory assessment.

### 2.2. Materials

Evaluation of IAP, TS, and CPM involved thermal stimuli being administered by a 30 × 30 mm thermode (PATHWAY, Medoc, Israel) to the centre of the right calf. The baseline temperature for stimuli used in all testing was 32°C, and the ramp up rate was 8°C/s. For CPM and TS, participants verbally rated pain intensity on a Numerical Rating Scale (NRS) ranging from 0 (“no pain”) to 10 (“extremely painful”). For IAP, participants provided verbal ratings indicating “to what degree were your thoughts/feelings about pain or something else,” using a 4-point Likert scale provided on paper (2 = “only pain,” 1 = “mostly pain,” −1 = “mostly something else,” and −2 = “only something else”).

### 2.3. Intrinsic attention to pain

Participants received 10 thermal stimuli (5/10 NRS—calibrated as a 20-second stimulus rated between 4 and 6—if a rating was given outside these values, the stimulus was adjusted by 0.5°C and the test was repeated) of 20 seconds(s) duration each and each with a 30-second interstimulus interval. After each stimulus, participants provided IAP ratings. IAP scores were calculated by averaging all 10 scores.

### 2.4. Temporal summation

Based on previous paradigms,^18^ a calibrated (5/10 NRS) thermal stimulus was applied for 120 seconds. Every 10 seconds, participants were verbally prompted to state pain intensity using the NRS. TS scores were calculated by subtracting the first score from the last score in the series. A high TS score indicated greater sensitization.

### 2.5. Conditioned pain modulation

Based on previous paradigms,^22^ the test stimulus was a calibrated 6/10 thermal stimulus applied to the right calf. The conditioning stimulus was submersion of the left hand in a 46.5°C water bath (Julabo, TW20). The test stimulus was first applied in isolation, with pain ratings from the leg recorded 3 times, at 10 seconds intervals over a 30 seconds stimulus. After this, the hand was submerged in the water bath, with 3 pain ratings from the hand recorded at 10 seconds intervals over a 30 seconds stimulus, checking everyone rated the conditioning stimulus as nonzero (ie, painful). Finally, the test and conditioning stimuli were presented simultaneously with pain ratings from the leg recorded 3 times, at 10 seconds intervals over a 30 seconds stimulus. The CPM score was calculated by subtracting the average pain rating from the test (leg) stimulus during simultaneous presentation, from the average rating from the test stimulus only condition. A high CPM score indicated more efficient inhibition of pain. Four participants were unable to keep their hand in the bath for the entire task, so final analysis includes the data of 40 participants.

### 2.6. Analysis

Regression models were used to explore whether IAP was associated with CPM and TS both individually and modelled together. For visualisation purposes, we calculated a composite “pronociception” score by adding TS scores (high facilitation) to reverse coded CPM scores (low inhibition), following z-transformation of both scales (the IAP scale was not z-transformed). A Pearson's correlation analysis was performed to explore the association between IAP and pronociception score. All statistical analyses were performed using SPSS 25 (IBM Corp, Armonk) with the significance level set to *P* < 0.05.

## 3. Results

Following a Cooks distance analysis removing 3 outliers,^3^ Table 1 shows that TS (t(36) = 11.08, *P* < 0.001) and CPM (t(36) = 4.91, *P* < 0.001) were significant. IAP was positively correlated with TS (r(35) = 0.36, *P* = 0.008) and negatively correlated with CPM (r(35) = −0.54, *P* = 0.001). The correlation between CPM and TS was nonsignificant (r(35) = 0.22, *P* = 0.187). The regression model showed that TS and CPM explained 39% of the variance in IAP scores (F(2,34) = 10.98, *P* < 0.001, *r* = 0.63, *R*^*2*^ = 0.39). CPM and TS remained significant predictors within the model (Table 2). Zero order and partial correlations within this model were roughly equivalent, indicating that the 2 measures explained unique portions of the variance in IAP.

**Table 1:** Statistics showing first and last rating of temporal summation (TS).

	Mean	SD	T	*df*	*P*	Correlation with IAP (*r*)	*P*
TS1 (initial rating)	2.46	1.61	11.08	36	0.000	0.01	0.943
TS12 (last rating)	7.24	2.06				0.56	0.000
CPM—average rating (test stimulus alone)	5.09	1.75	4.91	36	0.000	0.08	0.632
CPM—average rating (test and conditioning stimulus)	3.88	1.86				−0.54	0.001

Paired samples *t* test to show difference between first and last pain rating in TS paradigm. Correlation between IAP and first and last TS rating. For conditioned pain modulation (CPM), descriptive stats showing leg ratings for the test stimulus alone, and for test stimulus, when conditioning stimulus was added. Paired samples *t* test to show difference between test only and test + conditioning paradigm. Correlations between IAP and test only and test + conditioning paradigm.

IAP, intrinsic attention to pain.

**Table 2:** Intrinsic attention to pain (IAP) regression with conditioned pain modulation (CPM) and temporal summation (TS).

	B	SE B	β	T	*P*	Zero order	Partial
Constant	0.34	0.26		1.28	0.210		
CPM	−0.25	0.08	−0.47	−3.41	0.002	−0.54	−0.46
TS	0.10	0.04	0.33	2.39	0.023	0.43	0.38

**Figure 1.**
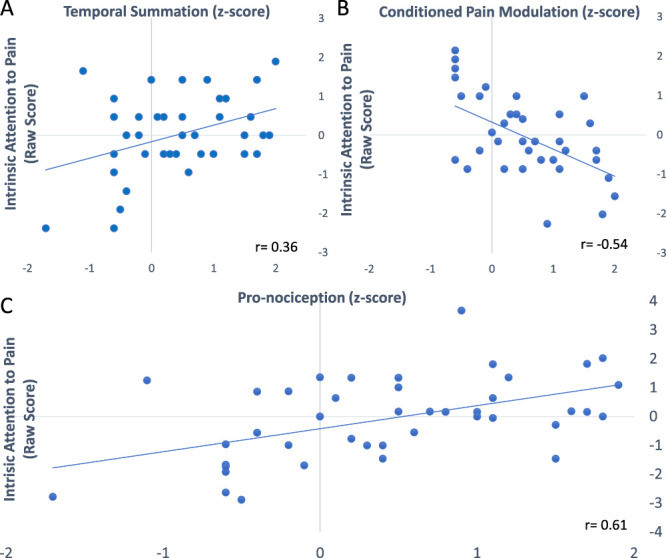
Scatterplots showing IAP correlations with temporal summation (A), conditioned pain modulation (B), and pronociception score (C). IAP, intrinsic attention to pain.

The pronociception score was correlated with the IAP score (*r* (35) = 0.61, *R*^*2*^ = 0.37—Figure 1). This correlation was consistent with variance explained in our regression model.

To check for robustness, the regression model was rerun including outliers (F(2,37) = 8.47, *P* < 0.001, *r* = 0.56, *R*^*2*^ = 0.31). IAP and TS were significantly correlated (r (38) = 0.43, *P* = 0.023), as were IAP and CPM (r (38) = 0.50, *P* = 0.001).

## 4. Discussion

This study examined the degree to which attention to pain is associated with endogenous mechanisms that modulate incoming sensory signals. We found an individual's IAP was associated with both TS and CPM. Both mechanisms seem to contribute to pain engagement relatively independently of each other, together accounting for 39 percent of the variance of attention to pain. This suggests modulation of spinal/supraspinal pain signals does significantly influence how we attend to pain, but other factors (eg, higher order cognitive processes) may also contribute strongly, as 61 percent of the variance in IAP is left unaccounted for.

Temporal summation occurs when a high frequency of action potentials in the presynaptic neuron elicits postsynaptic potentials that summate with each other,^5^ increasing pain perception. A previous study found that this windup was associated with activation of the ipsilateral and contralateral thalamus, medial thalamus, S1, bilateral S2, mid insula and posterior insula, rostral, and midanterior cingulate cortex,^17^ regions that have been previously implicated in attention studies.^11,12,19^

Less efficient CPM was also associated with IAP. CPM is strongly influenced by descending inhibitory signals from brainstem regions such as the periaqueductal gray.^1,4,7,20^ Periaqueductal gray activity has also been linked with IAP.^8^ This suggests the observed correlation may be related to overlapping mechanisms in the brainstem. Given that CPM explained only part of the variance in IAP, however, our findings are consistent with previous research indicating that distraction explained variance above and beyond CPM in explaining pain inhibition.^13^ This indicates that multiple factors influence attention. These may include evaluative cognitions involved in decision-making, assessment of risk/reward vs pain, or punishment avoidance.^2^

Yarnitksy acknowledges a “pronociceptive phenotype” consisting of 2 QST measures may not present a complete image of an individual's vulnerability to pain and could be supplemented with additional measures to achieve more precise characterization.^21^ These results support this suggestion by demonstrating that propensity to attend to pain is a function of processes such as windup and DNIC included in the “pronociceptive phenotype”, but these factors do not fully explain individual differences in IAP. Given that an individual's propensity to attend to pain is directly related to their ability to maintain cognitive function while experiencing pain, further investigation of the factors that contribute to IAP will provide clinically relevant clues as to why some individuals are able to maintain adaptive function despite living with pain.

## Disclosures

The authors have no conflicts of interest to declare.
